# Atrial fibrillation in a pediatric patient caused by an unusual malignant etiology: A case report

**DOI:** 10.3389/fped.2023.1051041

**Published:** 2023-02-23

**Authors:** Jelena Hubrechts, Christophe Vô, Cécile Boulanger, Katherine Carkeek, Stéphane Moniotte

**Affiliations:** ^1^ Division of Congenital and Pediatric Cardiology, Department of Pediatrics, University Hospital Saint-Luc, Brussels, Belgium; ^2^ Division of Pediatric Oncology, Department of Hemato-Oncology, University Hospital Saint-Luc, Brussels, Belgium; ^3^ Division of Neonatology, Department of Pediatrics, University Hospital Saint-Luc, Brussels, Belgium

**Keywords:** atrial fibrillation, child, lymphoma, cardiac involvement, pericardial invasion, cardioversion

## Abstract

This case report describes a 15-year-old patient with a known congenital malformation syndrome and immune deficiency, presenting with new-onset atrial fibrillation (AF) after a recent diagnosis of an intrathoracic mass. Transthoracic echocardiography showed a structurally and functionally normal heart and workup confirmed a primary diffuse large B-cell lymphoma, with pericardial and left atrial involvement on cardiac magnetic resonance imaging. Electrical cardioversion was successfully performed to convert the AF and chemotherapy was promptly started. Antiarrhythmic treatment was continued for 6 weeks, without recurrent AF. We discuss the pathogenesis of AF in the setting of malignancies as well as the management strategies of AF, mainly based on adult guidelines.

## Introduction

Atrial fibrillation (AF) is uncommon in children in the absence of congenital heart disease (1, 2). Epidemiological pediatric data are scarce and management is guided by studies performed in the adult population (3). The presence of AF in a young patient with a structurally normal heart requires a careful etiological workup. This case illustrates a rare malignant etiology of AF, an intrathoracic non-Hodgkin lymphoma with cardiac involvement, demonstrated on cardiac magnetic resonance imaging (MRI). To our knowledge, this is the first pediatric case described with new-onset AF caused by a neoplastic invasion of the left atrial wall.

## Case description

A 15-year-old girl, diagnosed with Hay–Wells syndrome–like phenotype at birth, was hospitalized for persistent cough, fatigue, and a deteriorating general condition. Her syndromic clinical features at birth and in infancy included cleft lip, cleft palate, maxillary hypoplasia, patchy alopecia, ankyloblepharon filiforme adnatum, absent eyelashes, dystrophic nails, bilateral syndactyly of the second to the fourth toe, ectrodactyly of both thumbs, hyperkeratosis, and hypoplasia of external genitalia. Furthermore, her medical history was marked by severe growth failure, celiac disease, gastroesophageal reflux, and hypogammaglobulinemia. She had no history of cardiac symptoms nor signs prior to this admission and there was no known family history of AF.

**Figure 1:**
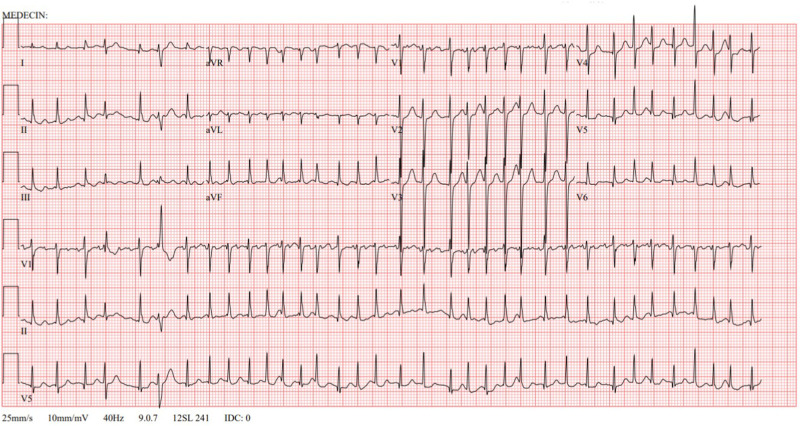
Electrocardiogram at onset. Atrial fibrillation with a rapid ventricular response. Incomplete right bundle branch block secondary to fast heart rate.

A persistent pleural effusion, found and monitored on chest x-ray and positron emission tomography computed tomography (PET-CT) scan, was suggestive of a malignant intrathoracic process. Primary mediastinal non-Hodgkin lymphoma was confirmed at anatomopathological analysis, with the definitive diagnosis of a stage III diffuse large B-cell lymphoma. Shortly after diagnosis and before starting chemotherapy and corticosteroids, the patient presented with acute onset chest discomfort and palpitations. A cardiac monitoring showed a heart rate varying between 200 and 220 beats per minute (bpm) with normal blood pressure for age. On electrocardiogram (ECG), AF was diagnosed, with a rapid ventricular response and incomplete right bundle branch block secondary to the fast heart rate (Figure 1). On previously performed transthoracic echocardiography (TTE), a small atrial septal defect–type secundum and normal biventricular dimensions and function were seen. Previous ECG was also normal with no signs of pre-excitation or early repolarization.

At the time of the acute AF, blood gas excluded electrolyte abnormalities. Hematologic analysis showed a hemoglobin level of 9 g/dl [Normal (N): 11–14.5 g/dL], hematocrit of 29% (N: 35%–47%), 3,000/mm^3^, neutrophils (N: 1,700–5,700/mm^3^) among 4,200/mm^3^ of white blood cells (N 4,000–10,000/mm^3^), and 489,000/mm³ of platelets (N: 1,75,000–3,45,000/mm^3^). Renal function and thyroid hormone levels were normal. Given her clinical state and hemodynamic tolerance, she was given an oral loading dose of amiodarone 800 mg/m^2^. Within 6 h, her ventricular response rate had dropped to 180 bpm, but she remained in AF and not in sinus rhythm. Over the next few hours, the clinical signs of increasing pallor and hepatomegaly developed and her blood pressure decreased to 79/43 mmHg. TTE showed normal cardiac function [with an ejection fraction (EF) of 70% and a shortening fraction (SF) of 39%], without enlargement of the left heart structures and no mitral valve regurgitation.

TTE was completed with a transesophageal echocardiography in order to rule out the presence of an intracardiac thrombus. Due to clinical deterioration, she was sedated (with propofol), intubated, and ventilated prior to electrical cardioversion. She returned to sinus rhythm after one shock of 1 J/kg. Anticoagulation by low-molecular-weight heparin (tinzaparin, 4,500 UI/day subcutaneously) was started and discontinued after 24 h of persistent sinus rhythm, without continuation of oral anticoagulation.

**Figure 2:**
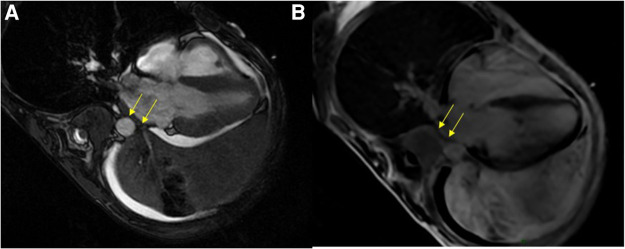
Cardiac MRI. SSFP four-chambers view showing the extensive tumoral process in the left pulmonary area coming into close contact with the left atrium, with paraneoplastic pleural and pericardial effusions (**A**); and likely tumoral invasion of the left atrial wall on the late-gadolinium-enhanced acquisition (**B**). MRI, magnetic resonance imaging; SSFP, steady-state free precession.

Etiological investigation was completed by performing cardiac MRI, which showed tumoral invasion into the pericardium and the lateral wall of the left atrium (Figure 2). Repeat TTE could not reproduce this finding.

Neurological examination at all times (at presentation and following treatment) remained normal. Cerebral MRI 24 h post-AF onset ruled out both intracranial tumoral invasion and signs of ischemic stroke.

Chemotherapy was started 24 h after AF onset according to the Inter-B-NHL 2010 treatment protocol. Amiodarone in maintenance dosage (200 mg/m^2^ 1×/day orally) was continued for 6 weeks without any use of antiarrhythmic agents thereafter. There was no recurrence of AF in this patient.

## Discussion

The incidence of AF in pediatrics is rare (prevalence <0.05% prior to the age of 30) and mainly documented in children with congenital heart disease, cardiomyopathy, inherited arrhythmias, hyperthyroidism, or post-open-heart surgery (2–4). Isolated AF, in the absence of underlying cardiovascular disease, represents less than 5% of all cases of AF, across all ages (1). Several risk factors for AF are described, such as hypertension, diabetes mellitus, obstructive sleep disorder, obesity, smoking, alcohol and drug use, excessive exercise, hyperthyroidism, or positive family history. The patient in this case report had none of these known risk factors (3, 5).

The pathogenesis of AF is generally multifactorial, with electrical remodeling, structural remodeling, and inflammation (6, 7). Once initiated, AF alters the electrophysiologic properties of the atrial myocardium, responsible for the maintenance of the arrhythmia. Furthermore, the role of a susceptible atrial anatomical substrate, such as myocyte degeneration, activation of fibroblasts, and enhanced connective tissue deposition leading to interstitial fibroses, has been implicated in the physiology of the onset and maintenance of AF. The presence of AF has also been associated with an increased inflammatory burden.

Invasion of the malignant tumor into the atrial wall myocytes in this case, is considered an inflammatory burden, creating a susceptible atrial anatomical structure, with the underlying milieu of the systemic proinflammatory state of cancer. In the absence of other underlying extracardiac triggers such as hypertension, hyperthyroidism, pulmonary embolism, viral infection, sepsis, or drug overdose, we considered the non-Hodgkin lymphoma with neoplastic invasion of the pericardium and the left atrial wall as the etiology AF in our patient.

The association between various cancers and AF has been described, justified by the abovementioned pathophysiological mechanisms of proinflammatory markers in both AF and cancer (8, 9). A large cohort study in a Danish population found that patients with new-onset AF had a markedly increased probability of neoplasia within 3 months following AF diagnosis, and that moreover, AF was strongly associated with metastatic cancer (10). Conversely, a recent meta-analysis suggests that patients with newly diagnosed cancer have a significantly increased risk of AF during the first 3-months of follow-up (9). Hospitalization costs, length of stay, and mortality rates are higher in cancer patients with AF than in those without AF. With regard to different cancer subtypes, in patients under the age of 65 years, AF has the highest association with lung cancer, followed by multiple myeloma and non-Hodgkin lymphoma (such as in our patient) (8).

Primary cardiac lymphoma is rare, and secondary cardiac involvement is even rarely reported. Usually, cardiac involvement is a late manifestation of lymphoma with median onset at 20 months after initial diagnosis, and often diagnosed at autopsy (11). The right side of the heart seems to be more often involved than the left and typically associated features include superior vena cava syndrome, pericardial effusion, and lymphatic return obstruction. Various arrhythmias can occur, including AF, atrioventricular block, and ventricular tachycardia (12).

Diagnostic modalities include CT and MRI, with contrast-enhanced MRI resulting in superior quality images to identify the morphology, location, and extent of intrathoracic masses (13).

Pediatric cases are very poorly described in the literature, and this patient reported is most likely the youngest to date reported with AF caused by pericardial and left atrial wall invasion of a non-Hodgkin lymphoma. Very few similar adult cases have been described (12, 14).

An added feature of note in this case is that during infancy, she was diagnosed with a Hay–Wells syndrome–like phenotype (15). The ankyloblepharon-ectodermal defects-cleft lip/palate (AEC) syndrome or Hay–Wells syndrome (MIM #106260) was first reported in 1976 (16). Hay–Wells syndrome belongs to a large, heterogeneous group of ectodermal dysplasia that affects the embryonic development of ectodermal tissues: hair, nails, teeth, sweat glands, and skin (17, 18). This very rare genetic condition is caused by a heterozygous mutation of the tumor protein p63 (TP63) gene, located on chromosome 3q28. Exome sequencing in our patient revealed *de novo* heterozygous variants in CHUK, PTGER4, and IFIT2. The variant in CHUK appeared to be most relevant for the AEC-like phenotype. CHUK is a direct target gene of p63 and encodes a component of the IKK complex that plays a key role in NF-*κ*B pathway activation (15).

As in most of the classic Hay–Wells patients, dermatological features were predominant in our patient. Cardiac features are extremely rare in these patients, with only ventricular septum defects and persistent ductus arteriosus described (19). No cardiac arrhythmias nor tumor development (such as lymphoma) have been reported in the literature linked to the syndrome. We did not consider her underlying genetic condition as the cause for her malignancy or AF.

With regard to the early workup in AF, early-stage cardiomyopathy and inherited ventricular arrhythmias should be ruled out/excluded. Rare channelopathies associated with AF in children include Brugada syndrome, long QT syndrome, and short QT syndrome. Baseline 12-lead ECG is a basic yet essential screening tool in the diagnosis and has high value in establishing prognosis and orienting further therapy (3). In our patient, a 12-lead ECG taken earlier was carefully examined using the Bazett formula, and no case for channelopathy was made out.

AF can also be associated with organized supraventricular tachycardia such as atrioventricular reentrant tachycardia degenerating into AF. Patients with accessory pathways have a much higher incidence of AF than the general population, especially with manifest accessory pathways (Wolff–Parkinson–White syndrome), but it has also been reported in patients with concealed accessory pathways (20).

AF is strongly associated with heart failure, even in the early stages of cardiomyopathy, and therefore (21), diastolic function on TTE should be carefully examined. Structural changes, however, may be delayed and develop later depending on the management, control, and re-occurrence of arrhythmias (3). In this case, TTE remained normal during follow-up.

Importantly, our patient did not receive any treatment in the form of anthracyclines, monoclonal antibodies such as ibrutinib, or other oncological treatments at the onset of AF. These medications are all considered risk factors for cardiovascular events (22–24).

In the current literature, treatment strategies for AF in children are not well defined. In general, rate control is often sufficient to resolve AF-related symptoms; however, rhythm-control strategies should always be considered in children. There is no robust evidence for the best choice of pharmacological agents offered for rate control, and the current practice includes the use of beta-blockers, calcium channel blockers, other antiarrhythmic drugs such as amiodarone or combination therapies.

Conversion to sinus rhythm by antiarrhythmic drugs is observed in approximately 50% in adults (7), and due to our patient's initial hemodynamic stability, we initially chose this strategy, in the knowledge that amiodarone appears to be more effective than sotalol in restoring sinus rhythm (25, 26). Pharmacological cardioversion also has the benefit of not requiring a fasting state or sedation.

In our patient, due to the inefficacy of pharmacological cardioversion and her worsening clinical state with hypotension, synchronized electrical cardioversion was subsequently the most appropriate procedure to rapidly establish sinus rhythm and avoid further clinical deterioration. Electrical cardioversion was performed despite the risk of stroke in non-anticoagulated patients (7) because it is indeed a quicker procedure and a more effective treatment of choice in hemodynamically compromised patients. It can be performed safely under sedation with intravenous midazolam and/or propofol. Continuous cardiorespiratory monitoring is essential.

Thromboembolism is a known complication of AF. The structural and functional changes of the atrial myocardium and stasis of blood generate a prothrombotic milieu. Anticoagulation is usually indicated in order to prevent stroke, although it remains controversial as there is a lack of pediatric recommendations. In adults, various risk scores exist to guide long-term anticoagulation decisions, such as the CHA2DS2-VASc score [congestive heart failure, hypertension, age ≥75 (doubled), diabetes, stroke (doubled), vascular disease, age 65–74, and sex (female)] recommended by the European Society of Cardiology (ESC) (7). Our patient was covered with low-molecular-weight heparin in view of electrical cardioversion, from the time of the procedure until 24 h thereafter. Continuous monitoring showed no recurrence of AF, and additional oral anticoagulation was not proposed in our patient, in accordance with adult guidelines (1, 7, 27).

The recurrent risk of AF is described between 15% and 39% after a first episode of isolated AF (4, 28). According to different case cohorts, age, male sex, obesity, and duration of the initial episode of AF were associated with a higher risk of recurrence. Throughout the treatment and follow-up of the patient's oncological condition, no recurrence was seen on monitoring. The indication for long-term antiarrhythmic therapy for AF has to be carefully balanced and weighed up, with negative AF-related symptoms on the one hand vs. the possible adverse effects on the other. Informed patient preference also plays a role (7). In cases of non-cardiac conditions associated with AF, the treatment of the underlying condition is crucial, as first-line therapy and (29) prognosis rely strongly on the underlying condition, but the lymphoma stage in our patient was unfortunately unfavorable.

In case of the recurrence of AF our patient, an electrophysiology study should be considered because other forms of supraventricular tachycardia that may trigger AF should be excluded (2, 29). Further future pediatric studies on this topic are needed in order to establish more specific guidelines on the management of AF in young patients. This study highlights the importance of clinical workup, examinations, and investigations to search for the cause of every new onset of AF in a pediatric patient.

## Conclusion

In young patients presenting with AF, with no prior history or findings of cardiac abnormalities, the investigative workup for determining etiology is vital. Oncological conditions, and especially intrathoracic or cardiac malignant lesions, are rare but a part of the differential diagnosis of new-onset AF, as demonstrated by this case report of a 15-year-old girl with non-Hodgkin lymphoma. Cardiac MRI is a helpful diagnostic tool and electrical cardioversion is a rapid and effective treatment option. Further pediatric studies are needed to establish clear management guidelines for isolated AF in pediatric populations.

## Data Availability

The original contributions presented in the study are included in the article/Supplementary Material, further inquiries can be directed to the corresponding author.
